# Cognitive Profile and Cardiovascular Risk Factors in Older Adults with Mild Cognitive Impairment

**DOI:** 10.3390/ijerph21040500

**Published:** 2024-04-18

**Authors:** Julia Vázquez-de Sebastián, Angel M. Ortiz-Zuñiga, Andreea Ciudin, Joan Ars, Marco Inzitari, Rafael Simó, Cristina Hernández, Sergio Ariño-Blasco, María José Barahona, Maite Franco, Xavier Gironès, María Cruz Crespo-Maraver, Joan Carles Rovira, Carmina Castellano-Tejedor, The DIALCAT Consortium

**Affiliations:** 1 Facultat de Psicologia, Universitat Autònoma de Barcelona (UAB), 08192 Barcelona, Spain; 2 RE-FiT Barcelona Research Group, Vall d’Hebron Institute of Research & Parc Sanitari Pere Virgili (VHIR-PSPV), 08023 Barcelona, Spaincarmina.castellano@uab.cat (C.C.-T.); 3 Vall d’Hebron Research Institute, Universitat Autònoma de Barcelona (VHIR-UAB), 08035 Barcelona, Spain; 4 CIBER de Diabetes y Enfermedades Metabólicas Asociadas, Instituto de Salud Carlos III Spain & Endocrinology Department, University Hospital Vall d’Hebron, 08035 Barcelona, Spain; 5 Medicine Department, Universitat Autònoma de Barcelona (UAB), 08192 Bellaterra, Spain; 6 Faculty of Health Sciences, Universitat Oberta de Catalunya (UOC), 08018 Barcelona, Spain; 7 Geriatric Service, Fundació Privada Hospital Asil de Granollers (FPHAG), 08402 Barcelona, Spain; 8 Endocrinology Service, Hospital Universitari Mutua Terrassa (HUMT), 08221 Terrassa, Spain; 9 Consorci Sanitari de Terrassa (CST), 08227 Terrassa, Spain; 10 Faculty of Health Sciences (UM-FUB), University of Vic-Central University of Catalonia, 08500 Vic, Spain; 11 Fundació Althaia-Xarxa Assistencial Universitària de Manresa (FA), 08243 Manresa, Spain; 12 Consorci Hospitalari de Vic (University Hospital of Vic), 08500 Vic, Spain; 13 GIES Research Group, Basic Psychology Department, Universitat Autònoma de Barcelona (UAB), 08192 Bellaterra, Spain

**Keywords:** cardiovascular risk factors, type 2 diabetes, cognitive impairment, neuropsychological profile, mild cognitive impairment, older adults

## Abstract

The prevalence of cardiovascular risk factors (CVRFs) in the older adults population and their specific impact on their cognitive profiles still requires further research. For this purpose, a cross-sectional study was carried out to describe the presence of CVRFs and their association with cognitive performance in a sample of older adults (65–85 years old) with Mild Cognitive Impairment (MCI). Participants (*n* = 185) were divided into three groups concerning their cardiovascular risk level determined by the presence of different CVRFs, including Type 2 Diabetes (T2D), dyslipidemia, hypertension, and obesity. The primary outcome measures were the participant’s scores in the Repeatable Battery for the Assessment of Neuropsychological Status (RBANS). Sociodemographic, clinical, and psychosocial data were collected. Non-parametrical statistical analyses and effect sizes were calculated. Findings revealed that a greater presence of CVRFs was not associated with a worse overall cognitive performance. High-risk patients were more likely to have significantly worse performance in the attentional domain compared to medium-risk (*p* = 0.029, *r* = 0.42) and compared to low-risk (*p* = 0.041, *r* = 0.35), specifically in the digits repetition subtest (*p* = 0.042). T2D alone was the CVRF associated with cognitive differences (*p* = 0.037, *r* = 0.32), possibly mediated by the duration of the condition. Consequently, a higher presence of CVRFs did not lead to a worse overall cognitive performance. However, high-risk individuals were more likely to experience cognitive impairment, particularly in the attentional domain. T2D played a significant role in these cognitive profile differences, possibly influenced by its duration.

## 1. Introduction

### 1.1. Cardiovascular Risk Factors (CVRFs) and the Aging Population

Cardiovascular diseases are the primary cause of mortality worldwide, markedly reducing quality of life [1]. Recent data from the global burden of Cardiovascular Diseases and Risks spanning from 1990 to 2022 highlight the significant impact of these conditions, resulting in 396 million years of life lost and 44.9 million years lived with disability [2]. In Spain, existing data reveal a steady increase in the prevalence of CVRFs among those over 65, with the most significant surge observed after age 75 [3]. Comorbidities and health complications associated with CVRFs, such as cognitive impairment—which is also closely related to aging—exacerbate the costs and impact on health care [4]. Consequently, the large number of people affected by these conditions poses challenges for health systems in managing this complex chronic disease. Identifying key factors in physical and cognitive decline, including modifiable lifestyle aspects, and eliciting affordable non-pharmacological interventions have become public health priorities [5].

### 1.2. Cardiovascular Risk Factors’ Impact on Cognition

CVRFs such as Type 2 Diabetes (T2D) [6], dyslipidemia [7], hypertension [8], and obesity [9,10] have been identified as predictive indicators of cognitive functioning and the risk of developing dementia. In this regard, research has indicated that a greater number of CVRFs are directly correlated with worse overall cognitive performance in a dose–response manner [11]. Consequently, some researchers emphasize the importance of focusing on the cognitive effects of overall risk rather than the impact of individual risk factors [12]. It is important to note that the timing of lifespan studies of sample populations plays a crucial role, as there appears to be a nonlinear, U-shaped association, with stronger risk estimates in mid-life compared to late-life [13]. However, this association remains unclear, with contradictory results, particularly for some factors, such as obesity [14,15]. For these reasons, it is crucial to continue expanding our knowledge about the cognitive impact of CVRFs, both collectively and in terms of their individual influence on older adults. This is particularly significant since factors such as T2D have shown more robust associations with the risk of developing dementia [16,17]. Among patients with T2D, higher glycemic levels and variables such as insulin treatment and duration since T2D diagnosis have been linked to these detriments [18,19,20]. Genetic predispositions carrying the APOE ε4 allele (Zhen et al., 2018) and/or the TOMM40 G allele (Gui et al., 2021), along with other factors such as a diagnosis of depression [18], high Body Mass Index (BMI) [18], and other cardiovascular comorbidities [20] have also been linked to exacerbating cognitive decline.

### 1.3. Cognitive Impairment-No Dementia Profile

Mild Cognitive Impairment (MCI), considered prodromal dementia, has demonstrated limited ability in predicting progression to dementia. Some authors argue for the need to distinguish the concept of MCI from cognitive impairment in patients with cardiovascular disease [21]. Consequently, Vascular Cognitive Impairment (VCI) was introduced to describe the spectrum of cognitive changes related to vascular causes, ranging from early cognitive impairment to dementia. Clinically, VCI can manifest as vascular dementia (either pure or combined with AD) or as Vascular Cognitive Impairment without dementia (i.e., VCI-ND), a prodromal condition associated with an increased risk of dementia [22]. Compared to MCI, VCI-ND has been associated with a lower risk of progression and a more stable course. However, more evidence is needed in this area, as this pattern is not always observed, and the associated cognitive changes associated can be variable.

Regarding the cognitive impairment profile of patients with CVRFs and T2D without dementia, some meta-analyses have shown the presence of performance deficits across multiple cognitive domains, with significant heterogeneity and contradictory results. Patients with vascular disease appear to exhibit poorer performance in executive functions [18], specifically in cognitive flexibility [23], working memory [19], and processing speed [19,24], detriments often associated with impaired fronto-cortical connections in cardiovascular patients rather than memory-related issues typically seen in the AD [17]. Other impaired domains include voluntary motor control and episodic and verbal or visual memory [23,24]. Nevertheless, these associations remain unclear due to overlapping cognitive profiles between vascular dementia, MCI, and AD cognitive profiles [25].

### 1.4. Justification for This Research

Despite all this evidence, the association of CVRFs with cognition has yet to be clarified. The study of mediating factors, such as clinical variables, genetic markers, sociodemographic data, and lifestyle indicators, will enhance our understanding of how CVRFs and T2D influence cognitive performance and its progression. A more comprehensive description of cognitive profiles in older adults with MCI (or Cognitive Impairment-no dementia; CI-ND) is crucial for improving predictive accuracy and developing more effective prevention and treatment strategies to mitigate CVRFs that could alter the disease’s course. Thus, a better understanding of cognitive disorders, based on recognizing these mechanisms and indicators will aid in developing new methods for their prevention and treatment [26].

This cross-sectional research was aimed to describe the presence of CVRFs (specifically, T2D, hypertension, hyperlipidemia, and BMI > 30 kg/m^2^) and study their association with cognitive performance in a sample of older adults with MCI (65 to 85 years old) to determine differences in impaired cognitive domains. The research questions related to the primary goal of this study were as follows:

(a) Is a greater number of cardiovascular risk factors associated with a worse overall cognitive performance in a dose–response manner in older adults with cognitive impairment-no dementia? (b) Are there differences in the cognitive profiles among the three levels of cardiovascular risk factors? (c) Is Type 2 Diabetes Mellitus the more robust factor associated with cognitive dysfunction?

Regarding the first research question, we hypothesized significant differences in overall cognitive performance among the three CVRF groups. Concerning the cognitive profile, we expected notable differences related to CVRFs between groups in attention, executive, and visuospatial functions, primarily, in non-amnestic functions. However, mixed results in research on memory functioning do not support a strong hypothesis. We anticipated that Type 2 Diabetes Mellitus would be the CVRF that most significantly influences cognitive performance.

## 2. Method

### 2.1. Study Design

A cross-sectional observational study was carried out (from September 2020 to June 2022) as part of a larger research project entitled DIALCAT Project: Diabetes as an accelerator of cognitive impairment and Alzheimer’s disease: comprehensive approach and adherence to treatment. DIALCAT was a multicenter, longitudinal study that included an observational prospective study and an mHealth randomized controlled trial (ClinicalTrials.gov Identifier: NCT03578991) conducted in different regions (Barcelona, Granollers, Terrassa, Vic, and Manresa) of Catalonia (Spain) to help T2D patients in improving their medication adherence and following up their physical and cognitive function. The duration of the project was three years (from January 2017 to January 2020). The DIALCAT project methodology is accessible at https://classic.clinicaltrials.gov/ct2/show/NCT03578991 (accessed on 1 April 2024).

### 2.2. Participants

The DIALCAT observational longitudinal total sample was *n* = 322. Participants were recruited from Endocrinology Departments and Geriatric Services from six reference healthcare centers in Catalonia (Spain) and invited to participate in the study when attending routine follow-up visits. Patients who voluntarily agreed to participate were included in the study after reviewing compliance with the inclusion/exclusion criteria. The subsample of 185 outpatient patients diagnosed with MCI was selected for this research.

Inclusion criteria: (a) Patients with T2D and non-diabetic aged 65 to 85 years (both included), (b) Patients diagnosed with MCI following the diagnostic criteria of the NIA-AA (Jack et al., 2018), (c) Patients showing ability to read and write in Spanish and/or Catalan. Exclusion criteria: (a) Family history of AD, (b) Mild-to-moderate AD, (c) Patients with other types of cognitive impairment, (d) Type 1 diabetic patients, (e) Advanced diabetic retinopathy (proliferative diabetic retinopathy, previous treatment with photocoagulation or intravitreal injections of anti-VEGF agents or corticosteroids), (f) Patients with severe uncorrected sensory deficits that make assessment impossible (blindness, deafness), (g) Patients with manifest language barrier related to the language of the scales and tests included in the study protocol (Catalan, Spanish), (h) Patients with premorbid intellectual disabilities, and (i) Patients with a recent personal clinical history (less than one year) of alcohol abuse or consumption of other illegal toxic substances.

Patients were divided into three groups concerning their cardiovascular risk level (CVRL) (low risk, medium risk, and high risk) based on the presence of different CVRFs, considering the following: T2D, dyslipidemia, hypertension, and BMI > 30 kg/m^2^. For the purposes of the present research, a CVRFs classification was developed based on cardiovascular risk prediction models, such as the Framingham Risk Score (FRS) [27], the SCORE (Systematic Coronary Risk Evaluation) [28], and the World Health Organization/International Society of Hypertension (WHO/ISH, Europe) models. Our CVRFs Calculator scores were as follows: The presence of T2D grants major risk with 3 points. BMI > 30 kg/m^2^, and hypertension and dyslipidemia grant 1 point each, resulting in 3 levels of CVRFs presence. The sum of all factors led to the following groups: 0–2, low risk; 3–4, medium risk; and 5–6, high risk.

### 2.3. Measures and Variables Collected

Sociodemographic and clinical data were extracted previously from patients’ medical records and confirmed with the patients’ responses in the hospital setting at their follow-up visit. Three health professionals participated in the visit. Firstly, the nurse performed blood testing. Secondly, one geriatrician or the endocrinologist collected the sociodemographic information using a clinical interview. Lastly, the neuropsychologist trained in its application performed the neuropsychological, functional, and psychosocial tests.

### 2.4. Primary Measures

Neuropsychological measures. The main outcome variables of this research were the global score and the standard index domain scores of the Spanish adaptation of the Repeatable Battery for the Assessment of Neuropsychological Status (RBANS) [29,30]. The RBANS is a short neuropsychological battery (administration time around 30 min, approximately) sensitive to detecting cognitive impairment in degenerative and non-degenerative pathology. It comprises 12 subtests divided into five domains: attention, language, visual-spatial/constructive ability, immediate memory, and delayed memory. The RBANS Form A was applied, for which Spanish normative data of the study population were available [31].

#### Secondary Measures

Sociodemographic and clinical data. Sociodemographics comprised the following: age, BMI, gender, educational level, and history of substance use (tobacco and alcohol). Clinical data comprised the following: diagnosis of T2D and years of evolution (years since T2D diagnosis), dyslipidemia, hypertension, obstructive sleep apnea syndrome, ischemic cardiopathy, presence of other cardiovascular illness, presence of peripheral arteriopathy, and other medical conditions. Also, a blood extraction was carried out using standard routines. The following laboratory parameters were considered: resting plasma glucose, glycosylated haemoglobin (HbA1c), triglycerides, total cholesterol, High-Density Lipoprotein cholesterol (HDL), and the allelic profile of the ApoE gene.

Psychosocial and functional data. The Clinical Dementia Rating Scale (CDR) [32]. is a widely used instrument to rate patients on the severity of dementia. Ratings are assigned by the clinician on a 0–5 point scale (0 = absent; 0.5 = questionable; 1 = present but mild; 2 = moderate; 3 = severe; 4 = profound; 5 = terminal) on each of the six domains of cognitive daily living performance: memory, orientation, judgment and problem-solving, community affairs, home and hobbies, and self-care. The information needed to perform each rating is obtained through a semi-structured interview of the patient and/or a reliable informant. The Barthel index [33], the Geriatric Depression Scale-short form (GDS-sf) [34], and other health status indexes, such as the EuroQol five dimensions questionnaire (EQ-5D) [35] and the Functional Social Support Questionnaire (DUKE-UNC) [36], were administered.

Procedure and Analyses. To pursue the objectives of this research, a part of the 2020-DIALCAT baseline database was employed. Statistical analyses of the data were carried out using SPSS 20.0 statistical software (IBM, Armonk, NY, USA). Descriptive data were reported as means (*M*), medians (*Mdn*), standard deviations (*SD*), ranges, and percentages (*%*) for all variables. Normal distribution was checked due to the small sample size before performing statistical tests. Since the vast majority of the outcome dimensions do not follow a normal distribution and the sample size is variable for the three groups, once classified in the three risk ranges, differences between groups based on the CVRFs Calculator (low, medium, high risk) and baseline measures were examined using the chi-square and the Kruskal–Wallis test and U-Mann–Whitney test, for qualitative and quantitative variables, respectively. Statistical significance was set at *p* < 0.05. Size effects were also calculated, including *Eta* (small effect < 0.01, medium effect 0.06–0.1, large effect > 0.1) for the chi-squared test and effect size *r* (small effect < 0.3, medium effect 0.3–0.5, large effect > 0.5) for U-Mann–Whitney test.

### 2.5. Ethical Aspects

All participants provided written informed consent, and the study protocol and procedures were approved according to the ethical standards of the Declaration of Helsinki and received approval from the Research Ethics Committees from all the participating institutions. All data were stored and saved anonymized in secure servers for scientific exploitation.

## 3. Results

### 3.1. Characteristics of the Sample

A total of 185 participants (*n* = 95 women, 51%) were included in the analyses with a mean age of 76 ± 6.1 years. Concerning clinical variables, 66.5% of participants (*n* = 123) had a T2D diagnosis, 71.7% (*n* = 132) had hypertension, and 67% (*n* = 124) had dyslipidemia. Regarding the ApoE genotype, a higher proportion of the participants were E4/E4 carriers (*n* = 110, 59.4%) versus E2/E4; E3/E4 carriers (*n* = 62, 33.6%) or E2/E2; E3/E3 (*n* = 13, 7%). All sociodemographic and clinical characteristics of the sample are presented in Table 1.

### 3.2. Comparisons between the Three Groups of CVRL

#### 3.2.1. Sociodemographic and Clinical Characteristics

Between-groups analysis has revealed statistical differences in age (*p* = 0.002). Comparisons of all pairs of groups showed that participants at high risk (*Mdn* = 74) were younger than participants at the other risk levels [high risk versus low risk (*Mdn* = 79), *U* (*N*_high risk_ = 90, *N*_low risk_ = 55,), = 1633,5, *z* = −3.434, *p* = 0.001, *r* = 0.98; high risk versus medium risk (*Mdn* = 77.5), *U* (*N*_high risk_ = 90, *N*_medium risk_ = 40,), = 1375,5, *z* = −2.145, *p* = 0.032, *r* = 0.40]. There were no statistically significant differences in gender or educational level between groups.

Since the groups were created based on CVRFs, several statistical differences in clinical characteristics between them were expected. Specifically, the groups differed in terms of BMI (*p* = 0.001), hypertension (*p* < 0.001), dyslipidemia (*p* < 0.001), and T2D (*p* < 001). In addition, participants at high cardiovascular risk had a greater time of evolution of T2D (*Mdn* = 15) than participants at medium risk (*Mdn* = 11), [*U* (*N*_medium risk_ = 26, *N*_high risk_ = 75,), = 692, *z* = −2.208, *p* = 0.027, *r* = 0.48]. Other statistical differences between groups were related to Obstructive Sleep Apnea Syndrome (*p* = 0.008) and peripheral arteriopathy (*p* = 0.021). No statistical differences between groups were found regarding alcohol consumption, smoking, ischemic cardiopathy, cardiovascular illness, and the APOE genotype.

#### 3.2.2. Psychosocial and Functional Measures

Significant differences between cardiovascular risk groups emerged concerning depressive symptoms (*H*(2) = 6.35, *p* = 0.042). In this sense, those patients within the high-risk (*Mdn* = 4) and low-risk groups (*Mdn* = 4), reported a greater number of depressive symptoms (higher scores in the GDS-15), compared to those at medium risk (*Mdn* = 3), [*U* (*N*_low risk_ = 55, *N*_medium risk_ = 39,), = 776.5, *z* = −2.286, *p* = 0.022, *r* = 0.54, *U* (*N*_medium risk_ = 39, *N*_high risk_ = 90,), = 1312.5, *z* = −2.286, *p* = 0.023, *r* = 0.46]. 

Regarding clinician rating of cognition, there were no differences in the scores of the CDR Scale (total, memory, orientation, judgment and problem-solving, community affairs, home and hobbies, and personal care), nor in the other psychosocial and functional measures, which were self-perceived social support (DUKE-UNC-15), self-rated health state (EQ-5D) and in the independence in activities of daily living (Barthel Index).

#### 3.2.3. Laboratory Parameters

Between-groups comparisons of the serum parameters are illustrated in Table 2. Participants differed according to their group of CVRL progressively. That is to say, participants at high risk showed worse levels of glucose concentration (*p* < 0.001), HbA1c (*p* < 0.001), and triglycerides (*p* < 0.001), followed by those at medium risk and those at low risk. Total cholesterol (*p* < 0.001) and HDL cholesterol (*p* < 0.001) levels turned out to be significantly different in an equally progressive but inverse manner; participants at low risk had worse serum parameters, followed by those at medium and high risk.

### 3.3. Research Questions

#### Neuropsychological Measures

RQ1. Is a greater number of cardiovascular risk factors associated with a worse overall cognitive performance in a dose–response manner in older adults with cognitive impairment-no dementia?

Table 3 includes the participants’ standardized scores in the RBANS test and the results of the Kruskal–Wallis test. A greater number of cardiovascular risk factors was not associated with a worse overall cognitive performance in the form of dose response, as there were no significant differences between groups in the RBANS Global Score.

RQ2. Are there differences in the cognitive profiles among the three levels of cardiovascular risk factors?

The Kruskal–Wallis test showed that the presence of CVRFs significantly affects attention performance (*p* = 0.034). Particularly, participants at high risk had significantly worse standard scores in the global attention score of the RBANS compared to participants at the other risk levels [*U* (*N*_low risk_ = 52, *N*_high risk_ = 87,), = 1793, *z* = −2.048, *p* = 0.041, *r* = 0.35, *U* (*N*_medium risk_ = 39, *N*_high risk_ = 87,), = 1284, *z* = −2.185, *p* = 0.029, *r* = 0.42]. When analyzing RBANS subtests, differences were seen in participants’ performance in the digits repetition task (*p* = 0.042), showing that participants at high risk had lower scores than participants at low risk and medium risk, this difference being significant just between high and medium risk participants [*U* (*N*_medium risk_ = 40, *N*_high risk_ = 89,), = 1346, *z* = −2.235, *p* = 0.025, *r* = 0.44].

RQ3. Is Type 2 Diabetes Mellitus the more robust factor associated with cognitive dysfunction?

Concerning the isolated effect of the CVRF (T2D, BMI, hypertension, and dyslipidemia), results indicated that T2D alone was directly associated with these cognitive profile differences. The Mann–Whitney test showed that the presence of T2D significantly affects attention tests performance, showing that participants with T2D score significantly less in the RBANS Attention Index (*Mdn* = 60) than participants without T2D (*Mdn* = 68), [*U* (*N*_no-T2D_ = 64, *N*_T2D_ = 122,), = 3180, *z* = −2.082, *p* = 0.037, *r* = 0.32]. There were no differences between T2D–noT2D participants’ performance in the digits repetition subtests.

## 4. Discussion

This study aimed to investigate the association between CVRFs and cognitive performance in a sample of older adults with MCI. In response to the initial research question, our results indicated that the presence of more CVRFs did not correlate with worse overall cognitive or functional performance in daily living activities. However, it is noteworthy that despite similar levels of impairment across the three groups, significant age differences were observed, suggesting that a higher co-occurrence of CVRFs might lead to earlier cognitive impairment. Younger participants at high risk paired their cognitive state with that of an older population, with a mean difference of 4 years before participants at the other risk levels. Notably, there were no significant differences in other demographic factors, such as gender and educational level. Therefore, our results align with existing studies that have shown that the presence of multiple CVRFs accelerates cognitive decline cumulatively, following a dose–response pattern [37,38]. Specifically, the literature has indicated that patients with T2D experience cognitive deficits at earlier ages (median of 75 years (65–86) versus 68 years (65–79)) [20]. However, it is important to interpret cross-sectional analyses between age and cognitive function with caution. The reason for these differences could be that older individuals tend to have fewer risk factors due to changes in their medications and weight loss, which are also associated with cognitive decline.

The effects of CVRFs on cognition have been extensively explored, but results have been inconclusive. This may be due to variations in sample characteristics (e.g., participants with dementia vs. participants without dementia, mid-life vs. late-life, lack of prior cognitive diagnoses), treatment status, and other mediating factors, including comorbidity. Thus, the potential for confounding is significant. In our study, we focused on analyzing the cumulative impact of different combinations of CVRFs in older adults with MCI. Concerning specific neuropsychological domains, our results revealed an additive effect of CVRFs on attention, with participants at higher risk demonstrating poorer overall attentional performance compared to those at lower risk levels. This effect may potentially be attributed to the extended duration of high-risk participants’ exposure to these factors. In particular, high-risk participants exhibited significantly impaired short-term verbal attention (digit repetition subtest) compared to medium-risk participants, whose performance was borderline. Given the cross-sectional nature of our study, these findings warrant further exploration through longitudinal prospective studies involving mid-life participants. Understanding how CVRFs influence cognitive decline over a lifespan is critical for developing effective preventive strategies for this vulnerable population. Previous longitudinal studies have already suggested an association between CVRFs and non-amnesic impairment in older adults [39,40]. In our sample, no significant differences were observed in other cognitive domains, although previous research has reported contradictory findings. Some studies, using the Framingham General Cardiovascular Risk Score and Magnetic Resonance Imaging, found an association between high cardiovascular risk and declines in global cognitive performance, executive functions, and vocabulary but not memory or visuospatial abilities [41,42]. In contrast, other studies have demonstrated associations with episodic memory, visuospatial abilities, and perceptual speed [43].

When examining the isolated effects of T2D, BMI, hypertension, and dyslipidemia, our results indicated a direct association between T2D and cognitive profile differences in attention. However, we urge caution in interpreting this finding due to the possible influence of confounding factors, as noted in previous cross-sectional studies involving older patients with T2D-MCI [44]. Gao et al. (2015) evaluated domain-specific effects of T2D on neuropsychological profiles, finding that performance in the digit span test was more impaired in individuals with T2D-MCI compared to controls. In contrast to our results, Gao et al. (2015) also reported detriments in executive and visuospatial functions, as assessed through block design and Trails B, as well as memory on the word learning list through delayed recall. Other cross-sectional studies examining neuropsychological effects on MCI patients found no differences in any test, including attention domain tests like the digit span, when comparing patients with and without T2D [45]. The discrepancies in these findings may be attributed to differences in cohort characteristics, such as community-based vs. clinical-based samples, as well as the interaction of various factors associated with T2D, including the duration of the condition, pharmacological treatment, and glucose monitoring, which may impact cognition. However, much of the existing literature on the effects of CVRFs, both in combination and isolation (e.g., Metabolic Syndrome and/or T2D), primarily focuses on no-dementia populations without specifying MCI diagnoses, thereby including participants with age-related cognitive decline. As a result, there is a need for further research specifically targeting MCI in older adults with various comorbidities.

In summary, this study reveals that MCI in older adults is more likely to manifest in individuals at higher risk and is characterized by poorer performance in the attentional domain. This population is also more susceptible to conditions like dementia, depression, and functional disabilities [45,46]. Cognitive dysfunction is especially significant due to its impact on quality of life. In older patients with T2D, cognitive dysfunction has been linked to inadequate diabetes control [47]. Therefore, it is crucial to monitor glucose control, adjust medications as needed, and provide safeguards against cognitive deficits through behavioral interventions [48].

Furthermore, it is important to note that the responsibility for the care of older MCI patients often falls on their families, resulting in significant personal and economic burdens. These costs have risen to $450 billion in the USA and $26 billion in Canada [49]. Evidence-based psychological techniques, part of what is known as Non-Pharmacological Interventions (NPIs), can enhance benefits and reduce the costs of interventions. Despite the growing interest in NPIs within the healthcare community, there is a lack of high-quality research on their implementation across various health conditions [50]. NPIs should encompass self-management, including medication adherence, treatment compliance, and daily glucose monitoring (in the case of T2D), as well as interventions to promote adherence to healthy lifestyles, such as regular physical activity and a nutritious diet. Research has shown that the most effective results are achieved through cognitive-behavioral (CB) methods [51,52]. These methods include stress management, relaxation techniques, self-monitoring of behavioral outcomes, problem-solving, cognitive restructuring, planning, goal-setting, modeling, self-efficacy, psychoeducation, and specific dietary and exercise recommendations. Additionally, new technologies like mHealth strategies, grounded in CB theories, have proven to be efficient in managing T2D in older adults [53]. New approaches rooted in well-established psychological theories are imperative, especially since the presence of CVRFs often correlates with mental disorders, potentially having multiplicative effects [54]. These findings are further supported by T2D research demonstrating the negative correlation of conditions like depression with treatment adherence [55]. Therefore, addressing overlapping risk factors through a multidisciplinary and integrative approach could expedite progress in managing these conditions and reducing associated disabilities [56].

This study must be considered within the context of several limitations. Firstly, the cross-sectional design used in this research does not establish causality. Additionally, it is important to acknowledge that our study participants were older adult outpatients with various CVRFs, making it inappropriate to generalize our findings to other population groups. Therefore, while the results may not be broadly applicable to other populations, they do provide insights into individuals with MCI between the ages of 65–85, who exhibit varying combinations of CVRFs and degrees of severity.

Future studies should delve deeper into the associations we have reported, particularly concerning the presence of CVRFs in diverse sample populations. One notable limitation is the absence of criteria tailored to diagnose MCI in vascular patients, as some MCI classifications fail to consider potential vascular factors contributing to cognitive impairment [57]. Incorporating vascular diseases into MCI diagnosis criteria could enhance clinical accuracy and our understanding of progression patterns [22]. Another potential source of bias arises from the original study design, which was initially intended for a different purpose than the one we pursued. Our sample represents a subset of a larger population, limiting the generalizability of our findings. Furthermore, due to variations in sample group sizes, non-parametric statistical analysis was employed. Additionally, the significant amount of missing data, as indicated in the results tables, may impact the robustness of our findings. Further research is essential to gain a comprehensive understanding of how CVRFs influence cognition in older adults with MCI. This knowledge is critical for mitigating the impact on the quality of life and impeding the progression of dementia by implementing appropriate interventions to effectively manage and reduce these modifiable risk factors.

## 5. Conclusions

In our sample of MCI older adults, the higher presence of CVRFs seems to determine worse cognitive status in the form of a severity gradient, not in the overall cognitive state but in the attention domain. These cognitive profile differences appear earlier in participants with a higher presence of CVRFs. Attention performance in older adults with composites of CVRFs may serve as a crucial predictive factor in the early stages of cognitive impairment. Findings suggest T2D alone is the only factor studied determining these differences, possibly mediated by the time of evolution of T2D. However, limitations inherent to cross-sectional multicenter studies with purposive samples make it imperative to research further how different CVRFs along the lifespan may influence MCI older adults’ profile and factors mediating this cognitive impairment.

## Figures and Tables

**Table 1:** Sociodemographic and baseline clinical characteristics of the sample (N = 185) according to the three levels of cardiovascular risk.

		Low Risk (*n =* 55)	Medium Risk (*n =* 40)	High Risk (*n =* 90)	Full Sample (*n* = 185)		
		*M (SD)*	*M (SD)*	*M (SD)*	*M (SD)*	*p* ^a^ *Effect Size*	
**Age (years)**		78 (5.6)	77.5 (5.7)	74 (6.1)	76 (6.1)	12.92; 0.002 *	1633.5; r = 0.98
**Body Mass Index** ^c^		27 (4)	28 (4.7)	31.1 (6)	29.2 (5.4)	23.2; <0.001 **	eta = 0.41
**Number of CVRF**		1.1 (0.8)	2 (0.6)	3.4 (0.5)	2.4 (1.1)	142.78; <0.001 **	eta = 0.77
		*n* (%)	*n* (%)	*n* (%)	*N* (%)	*p* ^b^	*effect size*
**Gender**	Female	31 (56.4)	21 (52.5)	43 (47.8)	95 (51.4)	1.034; 0.596	
	Male	24 (43.6)	19 (47.5)	47 (52.2)	90 (48.6)		
**Educational level** ^d^	Preschool education	10 (18.2)	8 (20.5)	25 (28.7)	43 (23.8)	16.66; 0.275	
	Primary education	25 (45.5)	14 (35.9)	30 (34.5)	69 (38.1)		
	Low secondary education	13 (23.6)	6 (15.4)	13 (14.9)	32 (17.7)		
	High secondary education	3 (5.5)	5 (12.8)	14 (16.1)	22 (12.2)		
	Superior Technician	0 (0)	1 (2.6)	1 (1.1)	5 (2.8)		
	Tertiary education	0 (0)	3 (7.7)	2 (2.3)	5 (2.8)		
	University or postgraduate	1 (1.8)	1 (2.6)	1 (1.1)	3 (1.7)		
**Alcohol consumption** ^d^	No	44 (81.5)	28 (71.8)	71 (80.7)	143 (79)	1.571; 0.456	
	Yes	10 (18.5)	11 (28.2)	17 (19.3)	38 (21)		
**Tobacco use** ^e^	No	38 (70.4)	27 (69.2)	59 (66.3)	124 (68.1)	4.689; 0.321	
	Yes	0 (0)	3 (7.7)	3 (3.4)	6 (3.3)		
	Ex-smoker	16 (29.6)	9 (23.1)	27 (30.3)	52 (28.6)		
**T2D**		Low Risk (*n* = 55)	Medium Risk (*n* = 40)	High Risk (*n* = 90)	Full sample (*N* = 185)		
		*n* (%)	*n* (%)	*n* (%)	*N* (%)	*p* ^b^	*effect size*
	No	55 (100)	7 (17.5)	0 (0)	62 (33.5)	159.082; <0.001 **	eta = 0.93
	Yes	0 (0)	33 (82.5)	90 (100)	123 (66.5)		
	Years of evolution ^h^	-	13.5 (1.9)	16.7 (0.9)	15.9 (8.7)	4.873; 0.027 *	692; r = 0.48
**Hypertension** ^f^	No	26 (47.3)	18 (46.2)	8 (8.9)	52 (28.3)	32.623; <0.001 **	eta = 0.42
	Yes	29 (52.7)	21 (53.8)	82 (91.1)	132 (71.7)		
**Dyslipidemia**	No	30 (54.5)	23 (57.5)	8 (8.9)	61 (33)	46.090; <0.001 **	eta = 0.5
	Yes	25 (45.5)	17 (42.5)	82 (91.1)	124 (67)		
**Obstructive Sleep Apnea Syndrome** ^g^	No	50 (96.2)	35 (89.7)	66 (77.6)	151 (85.8)	9.708; 0.008 *	eta = 0.23
	Yes	2 (3.8)	4 (10.3)	19 (22.4)	25 (14.2)		
**Ischemic Cardiopathy**	No	48 (88.9)	35 (87.5)	70 (77.8)	153 (83.2)	3.664; 0.160	
	Yes	6 (11.1)	5 (12.5)	20 (22.2)	31 (16.8)		
**Cardiovascular Illness** ^h^	No	50 (92.6)	37 (92.5)	81 (92)	168 (92.3)	0.017; 0.992	
	Yes	4 (7.4)	3 (7.5)	7 (8)	14 (7.7)		
**Peripheral Arteriopathy** ^i^	No	53 (98.1)	38 (95)	76 (85.4)	167 (91.3)	7.752; 0.021 *	eta = 0.206
	Yes	1 (1.9)	2 (5)	13 (14.6)	16 (8.7)		
**Apoe Genotype** ^j^	E2/E2; E3/E3	2 (22.2)	2 (22.2)	5 (55.6)	9 (7)	2.290; 0.683	
	E2/E4; E3/E4	11 (25.6)	7 (16.3)	25 (58.1)	43 (33.6)		
	E4/E4	26 (34.2)	16 (21.1)	34 (44.7)	76 (59.4)		

*Note.* ^a^ Chi-squared test is used for categorical variables ^b^ Kruskal–Wallis test for quantitative variables. *p*-value (*p* < 0.05) indicates statistical differences between CVR levels. * *p* < 0.05. ** *p* < 0.001. ^c^ 15 missing values. ^d^ 4 missing values. ^e^ 3 missing values. ^f^ 1 missing value. ^g^ 2 missing values. ^h^ 8 missing values. ^i^ 56 missing values. ^j^ 22 missing values.

**Table 2:** Laboratory parameters of the sample (*n* = 185) according to the three levels of cardiovascular risk.

	Low Risk (*n* = 55)	Medium Risk (*n* = 40)	High Risk (*n* = 90)	*p Value*
	*M (SD)*	*M (SD)*	*M (SD)*	*p* ^a^
**Glucose concentration (mg/dL)** ^b^	93.5 (11.2)	135.7 (44.4)	151.2 (59)	<0.001 **
**HbA1c (%)** ^c^	5.6 (0.3)	6.8 (1.1)	7.5 (1.3)	<0.001 **
**Triglycerides (mg/dL)** ^d^	112.6 (47.4)	130.2 (60.6)	159.2 (109)	<0.001 **
**Total cholesterol (mg/dL)** ^b^	195.8 (44.8)	183.6 (41.8)	170.9 (39)	<0.001 **
**High-density lipoprotein cholesterol (mg/dL)** ^d^	58.7 (12.7)	56.1 (17.7)	49.2 (13.7)	<0.001 **

*Note.* ^a^ *p*-value according to Kruskal–Wallis test (statistical significance at *p* < 0.05) between CVR levels. ** *p* < 0.001. ^b^ 7 missing values. ^c^ 14 missing values. ^d^ 10 missing values.

**Table 3:** Neuropsychological characteristics according to three levels of cardiovascular risk (*n* = 185).

		Low Risk (*n* = 55)	Medium Risk (*n* = 40)	High Risk (*n* = 90)	*p* ^a^
		*M* (SD)	*M* (SD)	*M (SD)*	
**RBANS Immediate Memory Index** ^b^		70.2 (18)	74 (18.2)	70.2 (15.6)	0.427
	List learning ^c^	5.3 (3.3)	5.9 (3.5)	5.1 (3.1)	0.399
	Story memory ^c^	4.6 (2.7)	5.3 (3.1)	4.8 (2.5)	0.501
**RBANS Visuospatial/Constructional Índex** ^d^		85.3 (23.2)	83.8 (20.7)	80.2 (19.1)	0.276
	Figure copy ^b^	7.7 (4.5)	7.7 (4.7)	6.8 (4.1)	0.347
	Line orientation ^b^	6.6 (3.6)	5.9 (3.1)	5.5 (3.4)	0.204
**RBANS Language Index** ^b^		85 (14.7)	83.6 (15.7)	83.9 (13.4)	0.891
	Picture naming ^c^	8.3 (3.2)	8 (3.3)	8.2 (3)	0.953
	Semantic fluency ^c^	5.6 (3)	5.4 (2.7)	5.5 (2.5)	0.990
**RBANS Attention Index** ^e^		67.7 (17)	67.9 (15.5)	61.9 (16)	0.034 *
	Digit span ^c^	6.3 (2.7)	6.7 (2.8)	5.6 (2.6)	0.042 *
	Coding ^f^	4 (3.1)	3.5 (2.6)	3.1 (2.7)	0.213
**RBANS Delayed Memory Index** ^b^		66.5 (24)	69.5 (19.7)	70.1 (18.6)	0.430
	List recall ^e^	5.4 (3.1)	5.8 (2.7)	5.6 (2.8)	0.712
	List recognition ^b^	4.3 (2.8)	4.7 (2.8)	4.1 (2.8)	0.604
	Story recall ^g^	4.6 (3.1)	5 (2.5)	4.7 (2.4)	0.523
	Figure recall ^g^	6.3 (4)	6.6 (3.6)	7 (3.4)	0.623
**RBANS Global Score** ^d^		70 (17.6)	70 (15.5)	67.2 (14.4)	0.612
**Clinical Dementia Rating (CDR)**	Memory ^h^	0.83 (0.56)	0.77 (0.62)	0.75 (0.47)	0.612
	Orientation ^e^	0.44 (0.44)	0.34 (0.46)	0.38 (0.44)	0.372
	Problem solving ^h^	0.7 (0.59)	0.63 (0.54)	0.69 (0.48)	0.617
	Community affairs ^h^	0.58 (0.66)	0.46 (0.52)	0.49 (0.5)	0.726
	Home and hobbies ^h^	0.61 (0.68)	0.37 (0.54)	0.49 (0.57)	0.137
	Personal care ^h^	0.18 (0.48)	0.21 (0.41)	0.19 (0.52)	0.726
	Total Score ^h^	3.36 (2.71)	2.78 (2.5)	2.99 (2.27)	0.364

*Note.* ^a^ *p*-value according to Kruskal–Wallis test (statistical significance at *p* < 0.05) between CVR levels. * *p* < 0.05. Data are standard scores and scaled scores for RBANS (Indexes and global score) and RBANS subtests, respectively. Data are raw scores for CDR and MMSE. ^b^ 5 missing values. ^c^ 3 missing values.^d^ 8 missing values.^e^ 7 missing values. ^f^ 9 missing values. ^g^ 4 missing values. ^h^ 6 missing values.

## Data Availability

The data that support the findings of this study are available from the corresponding author upon reasonable request.
